# *Staphylococcus aureus* Carrying *mec*C Gene in Animals and Urban Wastewater, Spain

**DOI:** 10.3201/eid2005.130426

**Published:** 2014-05

**Authors:** M. Concepción Porrero, Aránzazu Valverde, Pedro Fernández-Llario, Alberto Díez-Guerrier, Ana Mateos, Santiago Lavín, Rafael Cantón, José-Francisco Fernández-Garayzabal, Lucas Domínguez

**Affiliations:** Universidad Complutense, Madrid, Spain (M.C. Porrero, A. Valverde, A. Díez-Guerrier, A. Mateos, J.-F. Fernández-Garayzabal, L. Domínguez);; Universidad de Extremadura, Cáceres, Spain (P. Fernández-Llario);; Universitat Autònoma de Barcelona, Bellaterra, Spain (S. Lavín);; Hospital Universitario Ramón y Cajal and Instituto Ramón y Cajal de Investigación Sanitaria (IRYCIS), Madrid (R. Cantón)

**Keywords:** Staphylococcus aureus, mecC gene, wild animals, Spain, mecA homolog, MLST, antimicrobial resistance, wastewater, sewage, effluents, urban

**To the Editor:** A new methicillin resistance mechanism gene, a divergent *mec*A homologue named *mec*C (formerly *mec*A_LGA251_), was recently described in *Staphylococcus aureus* (*1*). Methicillin-resistant *S. aureus* (MRSA) isolates carrying *mec*C have been recovered from humans, ruminants, pets, and other animals such as rats, seals, and guinea pigs (*1**–**3*). It has been suggested that *mec*C-carrying MRSA isolates might not be detected by using MRSA selective media (*4*). For *mec*C-carrying *S. aureus* isolates, cefoxitin MICs of 4–64 mg/L have been demonstrated (*1**–**2*,*4*), values that would normally include susceptible isolates, according to the epidemiologic cutoff value established by the European Committee on Antibiotic Susceptibility Testing (EUCAST; www.eucast.org). *mec*C-carrying *S. aureus* isolates have been classified as heteroresistant (*5*), and MICs can be affected by the drug-susceptibility testing method used (*1**,**5*).

These observations led us to retrospectively investigate the presence of *mec*C gene in a set of 361 *mec*A-negative *S. aureus* isolates collected during 2009–2012 (Table), independently of their susceptibility to cefoxitin. Isolates were recovered from healthy carriers in livestock (n = 39), from wild animals (n = 254), and from wastewater (effluents) from an urban sewage plant (n = 68). Specific amplification of the *mec*C gene was performed as described (*6*). The *mec*C-carrying *S. aureus* isolates were tested by broth microdilution using Microtiter EUST plates (Trek Diagnostic Systems, East Grinstead, UK) for susceptibility to benzylpenicillin, cefoxitin, chloramphenicol, ciprofloxacin, clindamycin, erythromycin, florfenicol, fusidic acid, gentamicin, kanamycin, linezolid, mupirocin, rifampin, sulfamethoxazole, streptomycin, quinupristin-dalfopristin, tetracycline, thiamulin, trimethoprim, and vancomycin. Additionally, susceptibility to oxacillin was determined by using microScan Gram Positive Combo panel 37 (Siemens, Erlangen, Germany). MICs were interpreted according to EUCAST epidemiologic cutoff values.

**Table:** Testing of *Staphylococcus aureus* isolates for presence of methicillin resistance mechanism gene *mec*C, Spain*

Isolate source	Year(s) of isolation	No. *mec*C-positive isolates	*spa* type	MLST	CC	Antimicrobial resistance profile
Livestock, n = 39						
Cattle, n = 5	2011	0				
Fattening pigs, n = 34	2009, 2011	0				
Wild animals, n = 254						
Eurasian griffon vulture, n = 2	2011	0				
Fallow deer, n = 2	2012	2	t11212	ST425	CC425	PEN, FOX
			t11212	ST425	CC425	PEN
Iberian ibex, n = 39	2009–2010	0				
Mouflon, n = 2	2009	0				
Red deer, n = 61	2009–2011	0				
Wild boar, n = 148	2009–2011	1	t11212	ST425	CC425	PEN, FOX
Urban wastewater, n = 68	2011	1	t843	ST2676	CC130	PEN, ERY

*MLST, multilocus sequence typing; ST, sequence type; CC, clonal complex; PEN, benzylpenicillin; FOX, cefoxitin; ERY, erythromycin.

*mec*C was detected in a total of 4 isolates from wild boar (n = 1), fallow deer (n = 2), and urban wastewater (n = 1); these isolates represent 1% of the 361 tested isolates. The 3 isolates recovered from animals were susceptible to all antimicrobial drugs tested other than β-lactams and to oxacillin (MICs 0.5–1 mg/L) but were resistant to penicillin (MICs 0.5–2 mg/L). Two of the isolates were resistant to cefoxitin (MICs 8 and 16 mg/L) and the third was susceptible (MIC 4 mg/L). The wastewater isolate was resistant to penicillin (MIC 2 mg/L) and erythromycin (MIC 16 mg/L) and susceptible to all other antimicrobial drugs tested, including cefoxitin (MIC 4 mg/L) and oxacillin (MIC ≤0.25 mg/L).

Previous studies have described *mec*C-positive isolates as susceptible to all antimicrobial drugs tested except β-lactams (*2**,**3*), although sporadic resistance to fluoroquinolones has been found (*4**,**7*). We additionally found erythromycin resistance in 1 *mec*C-carrying *S. aureus* isolate. For the 4 *mec*C-carrying *S. aureus* isolates we detected, MICs of oxacillin were interpreted as susceptible, and 2 isolates were susceptible to cefoxitin according to EUCAST guidelines, findings that agree with previous reports (*1**–**2**,**4*). Thus, *mec*C presence is not always linked to resistance phenotypes for cefoxitin or oxacillin; such unclear findings could hinder the detection of *mec*C-carrying isolates.

We further characterized the 4 *mec*C-carrying *S. aureus* isolates by *spa* typing and detection of Panton-Valentin leukocidin (PVL) toxin genes (*6**,**8*). Multilocus sequence typing (MLST) was performed according to Enright et al. (*9*) by using self-designed primers *arc* (down 5′-CGATTTGTTGTTGATTAGGTTC-3′), *tpi* (up 5′-CATTAGCAGATTTAGGCGTTA-3′), and *yqi*L (down 5′-GATTGGYTCACCTTTRCGTTG-3′). All 4 isolates were PVL negative. The 3 animal isolates were assigned to a new *spa* type (t11212) and to clonal complex (CC) 425 and sequence type (ST) 425 (Table). ST425 has been previously associated with *mec*C-carrying *S. aureus* isolates in cattle and humans (*1**–**2*); the animals we sampled were from a game estate and may have had contact with cattle and with urban wastewater. The wastewater isolate was assigned to *spa* type t843 and to a new allelic profile, ST2676, in CC130 (Table). ST2676 represents a single-locus variant of ST130 carrying a different allele for the gene *aro*E. MRSA isolates of CC130 have been associated with humans and animals (*1**–**4**,**6*). This result indicates that *mec*C-carrying *S. aureus* isolates can be found in urban wastewater, which may act as an environmental reservoir, as has been demonstrated for *mec*A-carrying *S. aureus* (*10*).

In conclusion, we detected the methicillin resistance mechanism gene *mec*C in nonclinical *S. aureus* isolates from animals and urban wastewater in Spain. Although our data indicate that the frequency of this resistance mechanism is low, this gene appears to be expanding to new areas. Prospective studies should be performed to evaluate epidemiologic changes and to analyze the genetic lineages that carry this resistance mechanism.
